# Effect of Combining Organic and Inorganic Fertilizers on the Growth of Hemp (*Cannabis sativa* L.) Plants and the Accumulation of Phytochemicals in Their Inflorescence

**DOI:** 10.3390/plants14101519

**Published:** 2025-05-19

**Authors:** Mariarosaria Sicignano, Romina Beleggia, Luisa del Piano, Tommaso Enotrio, Serafino Suriano, Francesco Raimo, Daniela Trono

**Affiliations:** 1 Council for Agricultural Research and Economics (CREA), Research Centre for Cereal and Industrial Crops, Via Torrino 3, 81100 Caserta, Italy; mariarosaria.sicignano@crea.gov.it (M.S.); luisa.delpiano@crea.gov.it (L.d.P.); tommaso.enotrio@crea.gov.it (T.E.); francesco.raimo@crea.gov.it (F.R.); 2 Council for Agricultural Research and Economics (CREA), Research Centre for Cereal and Industrial Crops, S.S. 673, Meters 25200, 71122 Foggia, Italy; romina.beleggia@crea.gov.it (R.B.); serafino.suriano@crea.gov.it (S.S.)

**Keywords:** compost, digestate, cardoon waste, spent mushroom substrate, biomass, phenolic compounds, tocopherols, carotenoids, terpenes, cannabinoids

## Abstract

The feasibility of using a combination of organic fertilizer with a reduced rate of chemical nitrogen fertilizer as an alternative to conventional inorganic fertilization was tested on the growth and biomass accumulation of hemp plants and the phytochemical accumulation in their inflorescences. To achieve this goal, a field experiment was set up with the following nine treatments: F0, no fertilizer; NPK, mineral fertilizer with 100 kg ha^−1^ nitrogen; C1, compost from solid digestate (50%) + cardoon-based spent mushroom substrate (50%); C2, compost from solid digestate (50%) + straw-based spent mushroom substrate (50%); C3, C4, C5, and C6, composts from solid digestate (50%, 67%, 75%, and 84%, respectively) and cardoon waste (50%, 33%, 25%, and 16%, respectively); SD, non-composted solid digestate. C1–C6 and SD were added to the soil, along with half the rate (50 kg ha^−1^) of chemical nitrogen fertilizer. Taking F0 as a reference, all fertilized treatments, except C6 and SD, showed a notable increase in plant growth and biomass accumulation in the stem, inflorescence, and whole plant. Among the organic treatments, the best growth performances were detected in C1 and C5, which reached, or even exceeded, that of NPK. Compared to F0, all fertilized treatments had high phenolic acid and flavonoid yields, while high carotenoid, tocopherol, terpene, and cannabinoid (mainly CBD) yields were detected in all fertilized treatments except C6 and SD. Among the organic treatments, C1 and C5 stood out for their highest phenolic acid, flavonoid, carotenoid, and tocopherol yields, while C1, C2, and C3 stood out for their highest terpene and cannabinoid yields, which, in both cases, reached, or even exceeded, those of NPK. Overall, our findings show that 50% replacement of inorganic nitrogen fertilizer with C1 to C5 composts may represent a cost-effective and environmentally safe alternative to conventional inorganic fertilization that can sustain the growth of hemp plant and the phytochemical accumulation in its inflorescences, thus promoting the use of this crop for fiber and bioenergy production, as well as for applications in food, nutraceutical, agrochemical, and cosmetic sectors.

## 1. Introduction

*Cannabis sativa* L. is a herbaceous plant of the Cannabaceae family, domesticated around 12,000 years ago in Central Asia, which progressively expanded worldwide [1]. Based on the concentrations of the psychoactive cannabinoid ∆^9^-tetrahydrocannabinol (THC) and the two non-psychoactive cannabinoids, namely, cannabidiol (CBD) and cannabigerol (CBG), five distinct chemotypes of *C. sativa* can be distinguished [2]. Among these, chemotypes with high (more than 0.3%) THC concentration are known as drug-type, whereas chemotypes with an extremely low (less than 0.3%) THC concentration and high (more than 0.5%) CBD or CBG concentrations are referred to as “hemp” or “industrial hemp” [2].

Industrial hemp has been cultivated globally for centuries, primarily for its fiber, which is used in the production of paper, textiles, and ropes, as well as for its seeds, which are used for the production of oil or as food ingredients [3]. However, due to its botanical association with marijuana, hemp cultivation was halted in the early 1950s and was only resumed in the 21st century when new rules were introduced to encourage the cultivation of low-THC cultivars [4]. Thus, new applications of hemp raw materials have emerged, e.g., in the construction, bioplastic, and biocomposite industries, as well as animal bedding; whereas the biomass, which is a waste product of hemp cultivation, is being successfully used as a source of energy, heat, and biofuels [4]. Hemp is also an attractive crop from an agronomic point of view as it is a high-yielding crop and is noticeably resistant to pests, diseases, and many types of weeds, which means that it can be cultivated with little or no pesticides and herbicides [5]. In addition, hemp roots are also able to accumulate large amounts of metals, which makes this crop suitable for phytoremediation [5].

In recent years, hemp inflorescences have garnered attention as a source of phytochemicals, such as non-psychoactive cannabinoids, terpenes, and flavonoids, which are the most abundant, and other less represented compounds, which include alkaloids, tocopherols, and phytosterols [6]. All these compounds have been shown to have positive effects on human health and are therefore of interest to the pharmaceutical, nutraceutical, cosmetic, and agricultural industries [7]. Therefore, to improve the phytochemical profile of hemp inflorescences, there has been a recent surge in research aimed at evaluating how this profile varies depending on various factors, such as cultivar, harvest time, agronomic practices, cropping year, and response to stresses.

In agricultural practices, organic amendments, such as compost and digestate, are frequently used to improve the physical, chemical, and biological characteristics of soil, such as pH, electrical conductivity, porosity, aggregate stability, moisture, and organic and nutrient content [8,9]. Composts and digestates have also shown significant effectiveness against various phytopathogenic bacteria, fungi, and nematodes, which can help reduce the risk of plant diseases and the use of pesticides [10,11]. Furthermore, turning organic waste into compost and digestate accords with the principles of the circular economy as it creates useful resources from materials that would otherwise be discarded. This helps to reduce waste and promotes nutrient cycling, returning essential nutrients to the soil.

Nutrients in compost and digestate are released gradually as organic matter decomposes; therefore, applying these amendments promotes soil fertility over time and helps to prevent nutrients from leaching into groundwater [12,13], thereby creating a favorable environment that leads to better plant growth and increased crop productivity and quality [8]. However, although the slow release of nutrients is adequate after longer periods of soil amendment, it is not sufficient for the crops’ immediate needs. Indeed, it has been shown that the use of organic amendments as a unique source of nutrients only slightly increases crop yield in the short term [14]. To overcome this problem, an effective strategy is to combine the application of organic amendments with inorganic nitrogen at levels lower than usual. This combination of fertilizers achieves appropriate nitrogen release and mineralization rates while minimizing nitrogen loss [15,16].

In recent decades, the increased use of organic amendments and the concomitant reduction in chemical inputs into the soil has become one of the most important management techniques for the cultivation of various crops, including medicinal plants, which have shown an increase in biomass accumulation and an improvement in the chemical composition of their essential oil when grown under an organic regime [17,18,19,20]. Despite this, the use of organic amendments is currently not a common practice in hemp cultivation.

In light of the above, the present study sought to verify the feasibility of using a combination of organic fertilizer with a reduced rate of inorganic nitrogen fertilizer as an alternative to conventional inorganic fertilization in the cultivation of hemp plants intended for fiber and bioenergy production, as well as for applications in the food, nutraceutical, agrochemical, and cosmetic sectors. This was accomplished by evaluating the impact of using different compost blends and a non-composted solid digestate, associated with a reduced rate of inorganic nitrogen, on the growth and biomass accumulation of hemp plants, as well as on the phytochemical profile of their inflorescences. To achieve this goal, spectrophotometric, high-performance liquid chromatography (HPLC), and gas chromatography–mass spectrometry (GC-MS) approaches were applied to examine the changes in the profile and content of phenolic compounds, carotenoids, tocopherols, cannabinoids, and terpenes in the inflorescences of the dioecious cultivar Eletta Campana.

## 2. Results

### 2.1. Morphological Traits and Biomass Accumulation in Hemp Plants Grown Under the Different Fertilization Treatments

**Figure 1:**
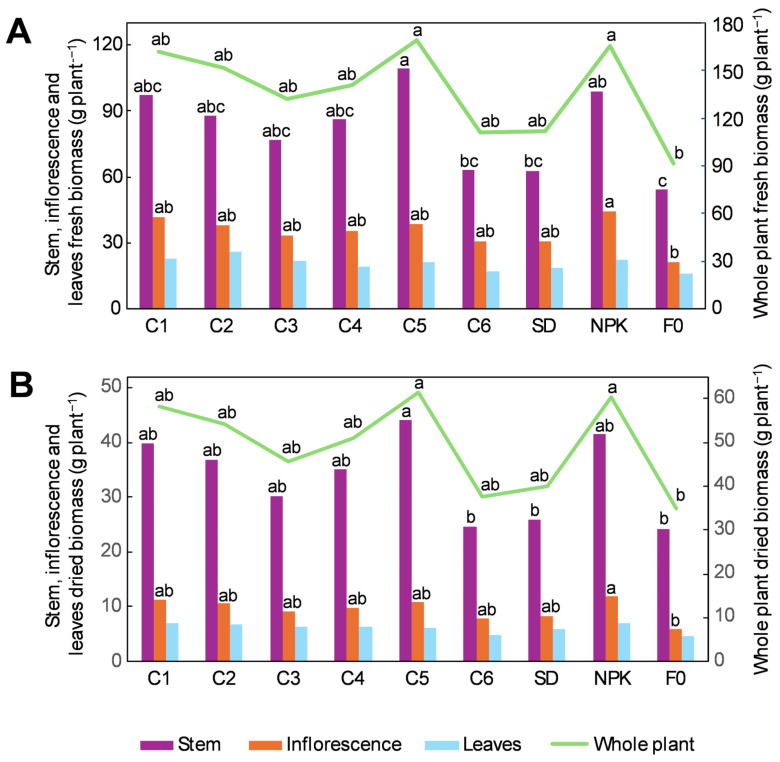
Effect of the different treatments on the fresh (**A**) and dried (**B**) biomass accumulation in the stem, inflorescences, leaves, and whole hemp plant. C1–C6, composts 1–6; SD, non-composted solid digestate; NPK, inorganic fertilization; F0, no fertilization. For each tissue, different lower-case letters represent significant differences among treatments (Tukey’s test *p* ≤ 0.05).

Differences in morphological traits and biomass accumulation were observed across the different treatments, although these were not always significant due to the high variability among the replicates (Table 1 and Figure 1). As shown in Table 1, C5 and NPK presented the greatest plant height gain compared to F0, with their values being 26% and 18% higher, respectively. Together with C1, C5 and NPK also presented the greatest gains in the other traits, showing a 28–36% larger basal stem diameter, a 32–42% larger middle stem diameter, and a 40–50% greater inflorescence length compared to F0. As for other treatments, C2, C3, and C4 showed slightly higher gains relative to F0 (ranging from +8% to +32%) than C6 and SD (from −8% to +17%). The number of leaves per plant did not change significantly among treatments.

The highest increases in fresh biomass were detected in C5 and NPK, followed by C1, which accumulated 102%, 82%, and 80% more fresh biomass in the stem, respectively, and 85%, 81%, and 77% more fresh biomass in the whole plant, respectively, compared to F0 (Figure 1A). A lower gain was observed in C2, C3, and C4, which, compared to F0, showed 42–62% more fresh biomass in the stem and 44–66% more fresh biomass in the whole plant, whereas only a small gain (from +15% to +22%) was observed in C6 and SD (Figure 1A). The accumulation of dried biomass followed a similar trend, with C5, NPK, and C1 showing an accumulation of 82%, 71%, and 65% more dried biomass in the stem, respectively, and 76%, 74%, and 68% more dried biomass in whole plant, respectively, compared to F0 (Figure 1B). In C2, C3, and C4, the dried biomass in the stem and in the whole plant exceeded that of F0 by 24–52% and 31–55%, respectively, whereas C6 and SD showed no increase compared to F0 (Figure 1B).

As for inflorescences, NPK had 107% and 104% more fresh and dried biomass, respectively, compared to F0 (Figure 1A,B). Although no significant differences were observed among the other treatments, the inflorescences of C1, C2, and C5 showed a greater gain compared to F0 than C3, C4, C6, and SD for both fresh (77–96% vs. 44–66%) and dried biomass (80–94% vs. 35–67%) (Figure 1A,B). Biomass accumulation in the leaves did not differ significantly among treatments, but a slightly higher accumulation (on average +33%) of fresh and dried biomass was observed in the leaves of fertilized treatments compared to F0 (Figure 1A,B).

### 2.2. Phytochemical Content in the Inflorescences of Hemp Plants Grown Under the Different Fertilization Treatments

The inflorescences of hemp plants grown under the nine treatments were analyzed for the total phenolic content (TPC), total flavonoid content (TFC), and total antioxidant activity (2,2-diphenyl-1-picrylhydrazyl, DPPH), determined spectrophotometrically, and the content of secondary metabolites, determined by HPLC and GC-MS. A total of 55 metabolites were detected, which included 8 phenolic acids, 8 flavonoids, 3 carotenoids, 3 tocopherols, 8 monoterpenes, 21 sesquiterpenes, and 4 cannabinoids.

#### 2.2.1. Phytochemical Profile Composition

**Figure 2:**
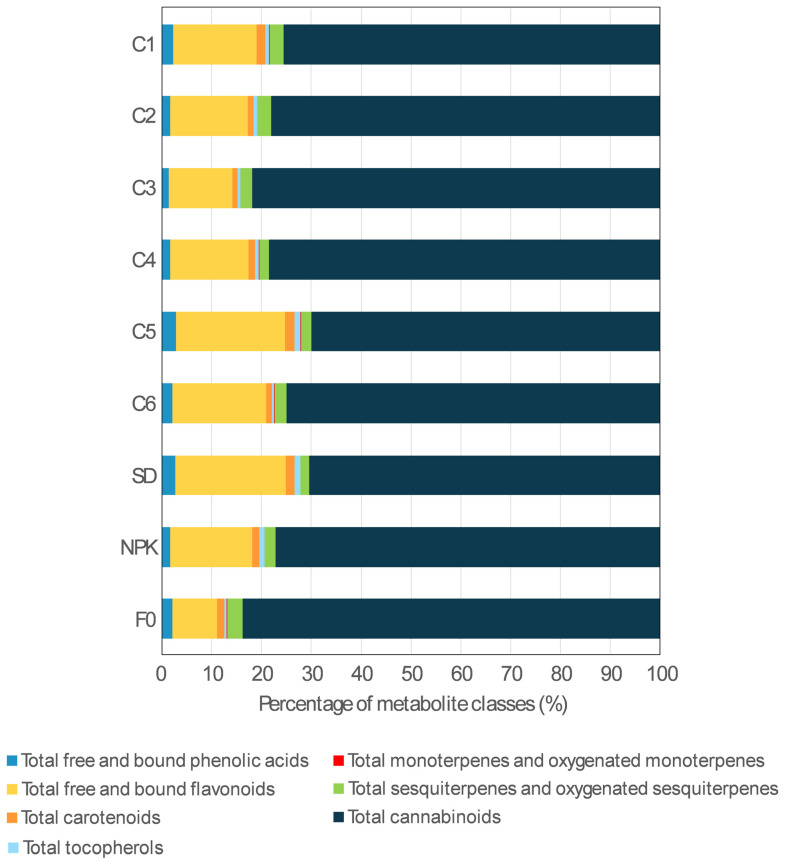
Composition of the secondary metabolite profile in the inflorescences of the hemp plants grown under the nine treatments. For each treatment, the percentage of each class of metabolites was calculated in relation to the sum of all the metabolites detected in that treatment. C1–C6, composts 1–6; SD, solid non-composted digestate; NPK, inorganic fertilization; F0, no fertilization.

Regarding the abundance of the different classes of secondary metabolites, cannabinoids and flavonoids were the two most represented classes across all nine treatments (Figure 2). As for cannabinoids, the profile of C3 and F0 presented the greatest contribution from this class of metabolites, which accounted for 81.8% and 83.7% of the total metabolite content, respectively, whereas the lowest contribution was detected in C5 and SD, with a percentage of 70.0% and 70.4%, respectively. Percentages of total cannabinoids ranging from 75.0 to 78.5% were detected in the other treatments.

The opposite trend was observed for flavonoids, with C5 and SD presenting the highest percentages (21.9% and 22.1%, respectively), whereas the lowest percentages (12.7% and 8.9%, respectively) were detected in C3 and F0. In the other treatments, the flavonoid contribution ranged between 15.6% and 18.8%. The percentage of total sesquiterpenes (hydrocarbons and oxygenated) ranged between 1.7% and 3.2%, with the greatest contributions detected in C1, C2, and F0, and the lowest in SD. The percentages of phenolic acids, carotenoids, and tocopherols were 1.4–2.9%, 1.1–1.9%, and 0.5–1.2%, respectively, whereas the percentage of monoterpenes (hydrocarbons and oxygenated) was below 0.1% in all treatments.

#### 2.2.2. Phytochemical Content

**Figure 3:**
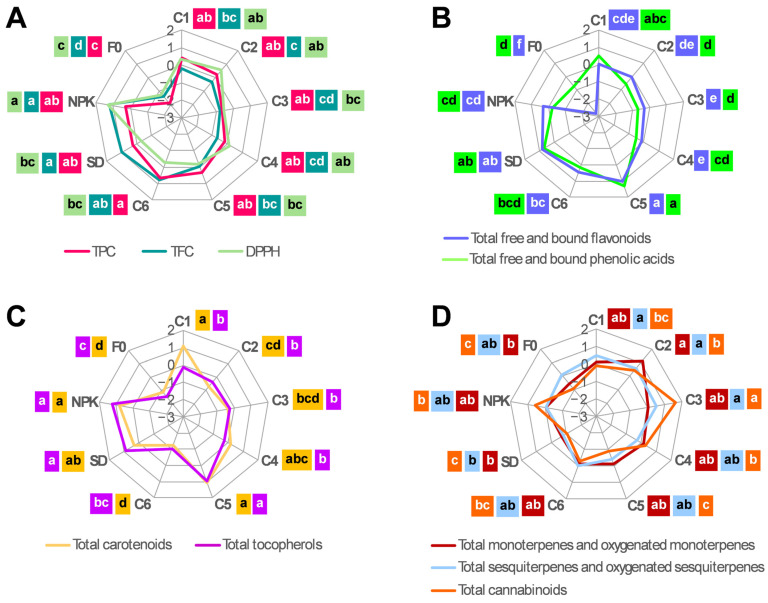
Radar charts comparing TPC, TFC, and DPPH (**A**), total flavonoid and phenolic acid content (**B**), total carotenoid and tocopherol content (**C**), and total monoterpene, sesquiterpene, and cannabinoid content (**D**) detected in the inflorescences of the hemp plants from the cv Eletta Campana grown under the nine treatments. To represent the traits on the same scale, values from each trait were normalized by subtracting the mean and dividing by the standard deviation. Tukey’s multiple test (*p* ≤ 0.05) was carried out for each class of metabolites, and the result obtained is reported with lower-case letters highlighted with the same color as the corresponding class. C1–C6, composts 1–6; SD, non-composted solid digestate; NPK, inorganic fertilization; F0, no fertilization; TPC, total phenolic content; TFC, total flavonoid content; DPPH, 2,2-diphenyl-1-picrylhydrazyl.

To provide an overview of how the nine treatments affected the accumulation of the different classes of metabolites, radar charts were used, normalizing the dataset on the same scale (Figure 3). The specific content of the individual metabolites is detailed in Table 2.

As for TPC, its trend resembled those of TFC and the total flavonoids detected by HPLC (Figure 3A,B); this was because flavonoids (determined both spectrophotometrically and by HPLC) accounted for the largest fraction (on average 40%) of TPC. F0 had the lowest TPC, TFC, and total flavonoid content (on average, −40%, −40%, and −61%, respectively, compared to the other treatments) (Figure 3A,B). Consistently, almost all the individual flavonoids were found at their lowest levels in F0, particularly the most abundant flavonoids, such as free vitexin, isovitexin, and orientin (on average, −68%, −67%, and −44%, respectively, compared to the other treatments) (Table 2). Although not always significant, higher total flavonoid content was detected in C5, C6, SD, and NPK compared to C1, C2, C3, and C4, with the highest level (on average, +21% compared to the other treatments) detected in C5 (Figure 3B). This was mainly due to free vitexin, which, in C5, was at higher levels (on average, +55%) compared to the other treatments (Table 2). C5 also presented the highest content of total phenolic acids (on average, +50% compared to the other treatments) (Figure 3B), which was mainly due to its higher levels of free and bound *p*-coumaric and ferulic acids (on average, +65%) compared to the other treatments. (Table 2).

As for antioxidant activity, F0 and NPK had the lowest and the highest levels, respectively (on average, −11% and +12%, respectively, compared to the other treatments) (Figure 3A). It is worth noting that the trend (shape of the curve) of antioxidant activity across treatments resembled those of TPC, TFC, and total flavonoids (Figure 3A,B). In line with this observation, a significant correlation was found between the antioxidant activity and TPC (r = 0.34; *p* = 0.047), TFC (r = 0.38; *p* = 0.028), and total flavonoids (r = 0.32; *p* = 0.046), but not with the other classes of metabolites, which indicated that the antioxidant activity in the hemp inflorescences of the cv Eletta Campana was mainly due to the flavonoid component.

As for total carotenoids and total tocopherols, the lowest values were detected in C6 and F0 (on average, −30% and −42%, and −27% and −57%, respectively, compared to the other treatments) (Figure 3C). Consistently, the most abundant carotenoids and tocopherols, namely, lutein, β-carotene, and α-tocopherol, accumulated at their lowest levels in C6 and F0 (on average, −37% and −41%, respectively, compared to the other treatments). Among the other treatments, higher levels of total carotenoids and total tocopherols (on average, +25% and +41%, respectively) were detected in C1, C5, SD, and NPK compared to C2, C3, and C4, which is in line with the higher accumulation (on average +34%) of lutein, β-carotene, and α-tocopherol in the former compared to the latter group of treatments (Table 2).

Total monoterpene levels were highest in treatment C2 and lowest in treatments SD and F0 (on average, +83%, −38%, and −28%, respectively, compared to the other treatments) (Figure 3D). This trend was consistent across almost all the individual monoterpenes, especially α-pinene, which also showed the highest level in C2 and the lowest level in SD and F0 (on average, +273%, −60%, and −35%, respectively, compared to the other treatments) (Table 2). Regarding the total sesquiterpenes, C1, C2, and C3 presented higher levels (on average +37%) compared to the other treatments (Figure 3D). This was consistent with the higher content of β-caryophyllene, α-humulene, β-curcumene, and α-bisabolol detected in C1 (on average +36% compared to the other treatments), as well as the higher levels of nearly all of the individual sesquiterpenes detected in C2 and C3 (Table 2).

As for total cannabinoids, F0, together with C5 and SD, presented the lowest levels (on average, −23% compared to the other treatments), whereas the highest level was detected in C3 (on average, +38% higher than the other treatments) (Figure 3D). This trend resembled that of CBD, which was found at lower levels in F0, C5, and SD (on average, −22%) and at higher levels (on average +23%) in C3 compared to the other treatments (Table 2).

### 2.3. Phytochemical Yield in Hemp Plants Grown Under the Different Fertilization Treatments

Given the significant effects of the treatments on both the dried biomass of the inflorescences and on their phytochemical content, the yield per plant of each class of phytochemicals was calculated. As shown in Table 3, all fertilization treatments gave higher yields compared to F0. Taking F0 as a reference, C1 and C5 had a gain comparable or even higher than NPK for phenolic acid (181% and 217%, respectively, vs. 135%), flavonoid (358% and 436%, respectively, vs. 402%), and carotenoid (204% and 186%, respectively, vs. 194%) yields. In the other treatments, the gain ranged between 60% and 103% for phenolic acids, 245% and 324% for flavonoids, and 23% and 118% for carotenoids. C5 also presented a gain in tocopherol yield comparable to NPK (523% vs. 580%), whereas for the other treatments, the gain ranged between 98% and 333%. For both carotenoids and tocopherols, the lowest gain was observed in C6. C1 and C2 had a higher gain compared to NPK for monoterpenes (227% and 354%, respectively, vs. 194%) and sesquiterpenes (130% and 120%, respectively, vs. 98%). As for the other treatments, C3, C4, and C5 presented a gain higher than C6 for both monoterpenes (128–155% vs. 62%) and sesquiterpenes (34–82% vs. 25%), whereas no gain was detected in SD. C1, C2, and C3 also had the highest cannabinoid gain, along with NPK (131–170%), followed by C4 and C5 (116% and 92%, respectively), whereas the lowest gain was detected in C6 and SD (52% and 37%, respectively).

### 2.4. Multivariate Analysis

**Figure 4:**
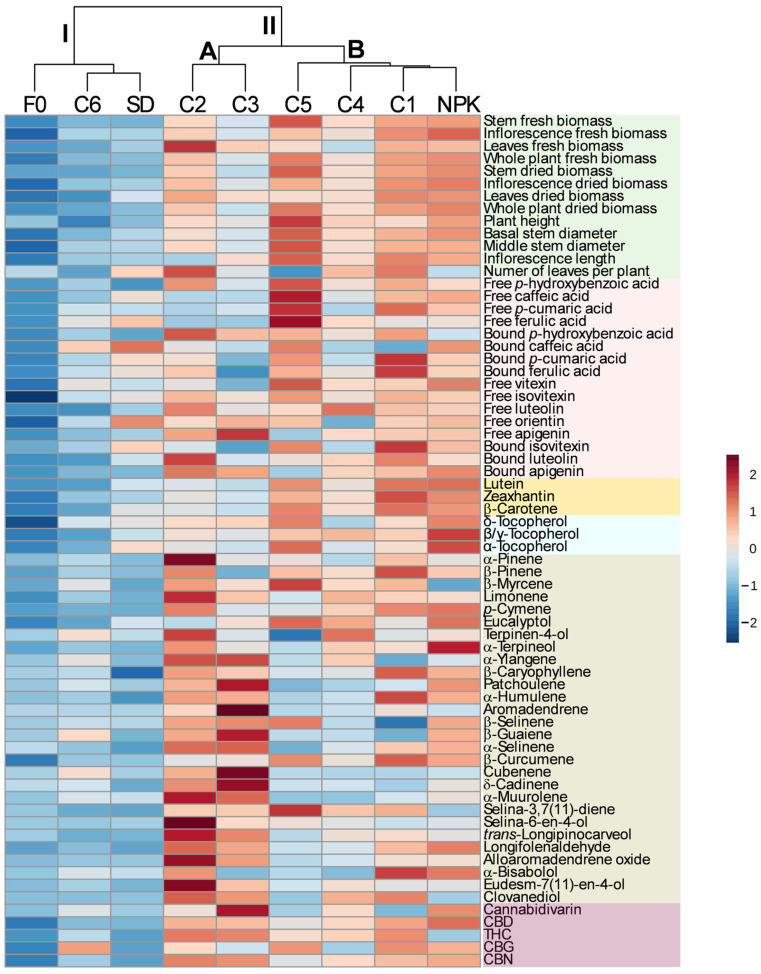
Hierarchical clustering analysis and heatmap of morphological traits, biomass accumulation, and yield per plant of the individual phytochemicals detected in the inflorescences of the hemp plants from the cv Eletta Campana grown under the nine treatments. C1–C6, composts 1–6; SD, non-composted solid digestate; NPK, inorganic fertilization; F0, no fertilization.

Hierarchical cluster analysis (HCA) combined with the heatmap was carried out on the dataset including all the traits related to plant morphology, biomass accumulation, and the yield per plant of the individual phytochemicals. The results obtained are reported in Figure 4. The HCA identified two clusters, cluster I and cluster II, with the latter including two subclusters, subcluster A and subcluster B. Cluster I included treatments C6, SD, and F0, which were characterized by the lowest growth performances and yield per plant of most of the individual phytochemicals. Subcluster A included treatments C2 and C3, which had intermediate growth performance and yield of phenolic compounds, carotenoids, and tocopherol, as well as high yield of terpenes and cannabinoids; whereas subcluster B included treatments C1, C4, C5, and NPK, which showed medium-to-high growth performances and yield of phenolic compounds, carotenoids, and tocopherol; C1 and NPK were distinguished from C4 and C5 by the higher yield of terpenes and cannabinoids.

## 3. Discussion

In the last decade, hemp cultivation has gained renewed attention due to its cultivation benefits and the potential for positive economic returns from its fiber, biomass, and phytochemicals. Despite this, our understanding of the best practices for hemp cultivation, especially with respect to organic production systems, still contains numerous gaps. Indeed, there are very few studies aimed at evaluating the effect of organic fertilization on hemp plant growth and yield [21,22,23], and only one of these has also evaluated the effect on the accumulation of CBD and THC [22]. The present study has filled this gap, at least partially, by revealing the ability of different composts and a non-composted solid digestate to affect the growth of hemp plants and the accumulation of phytochemicals in their inflorescence.

### 3.1. Effect of the Different Treatments on the Growth and Biomass Accumulation of Hemp Plants

The results reported in the present study revealed reduced growth and biomass accumulation in hemp plants cultivated under unfertilized conditions, thus confirming that adequate mineral fertilization is essential for the efficient growth of hemp plants. This is especially true for nitrogen fertilization, which influences the hemp growth and biomass yield more than phosphorus and potassium [24,25]. In the present study, 100 kg ha^−1^ of nitrogen was chosen as the full rate based on previous studies reporting that hemp plants grown under field conditions required a nitrogen rate between 80 and 140 kg ha^−1^ for the achievement of optimal growth and biomass yield [26,27]. In line with these observations, chemically fertilized plants exhibited enhanced growth and 74% higher accumulation of dried biomass in the whole plant compared to unfertilized plants.

Regarding the combined mineral and organic fertilization, the results obtained indicated that 50% replacement of inorganic nitrogen fertilizer with composts C6 and SD produce no great gain in plant growth and biomass accumulation compared to F0, while the combination with composts C1, C2, C3, C4, and C5 led to noticeable increases, although differences were observed among composts. Consistent with this observation, previous findings have highlighted the differential effects of different composts on soil, particularly in terms of its aggregates, microbial flora, and the availability of nutrients, and on plant nutrient uptake and growth [28]. The best performance was observed when mineral nitrogen was combined with composts C1 and C5. These combinations exhibited gains in plant growth and biomass accumulation comparable or even greater than those observed with the sole utilization of chemical fertilizer. Similarly, 25–30% replacement of synthetic nitrogen fertilizer by poultry manure or cattle waste compost maintained the same nitrogen uptake and biomass accumulation as conventional nitrogen fertilization in maize [29]. In tomato, the same fruit yield as conventional nitrogen fertilization was maintained when 40% of synthetic nitrogen fertilizer was replaced with manure or manure plus pruning compost [30], whereas an even higher biomass and seed yield was obtained in wheat by reducing the nitrogen rate by 50% through the addition of rock phosphate-enriched compost [31]. Hemp plant biomass is a waste product of hemp cultivation for food, cosmetic, or pharmaceutical applications, which can be successfully used for the production of liquid, gaseous, and solid biofuels [32]. Therefore, the combination of C1 or C5 compost, with half the rate of inorganic nitrogen, similar to the full rate of inorganic nitrogen, can be useful for obtaining hemp raw material to be used in the production of renewable energy. This promotes hemp waste reduction and the transition to a circular economy, which perfectly fits into the idea of sustainable development. In addition, C5 outperformed NPK for the dried biomass gain in the stem, making the use of this compost particularly suitable when the crop is intended for fiber and hurd production [32].

Compared to F0, composts C2, C3, and C4 also induced an increase in plant growth and biomass accumulation, but to a lesser extent than C1 and C5, with the lowest gain detected for compost C3. This could be explained by the nitrogen levels being the lowest in C3. Evidence suggests that low nitrogen content in the compost may limit plant growth because of microbial immobilization and the competition for nitrogen between soil microorganisms and plants [33,34]. However, other factors may also be involved since compost C6, which had a nitrogen level comparable to C5, was unable to stimulate the development of hemp plants. A possible explanation for this could be the lower rate of nitrogen mobilization in C6 compared to C5, which led to an inadequate nitrogen level in the soil. In this regard, Duong and coworkers [28] observed that composts made from similar feedstocks (i.e., organic fraction of solid municipal waste) and with the same nutrient content presented different abilities with respect to improving soil fertility and plant growth, and the authors ascribed this to different rates of nutrient release in the soil. Regarding treatment SD, its failure to stimulate hemp plant growth is most likely due to its high carbon content. Indeed, it was found that the high carbon level that characterizes solid—compared to liquid and whole—digestate stimulated soil microorganisms to proliferate and immobilize nitrogen from the soil, thus exhausting the nitrogen reserves available for plant [35].

### 3.2. Effect of the Different Treatments on the Phytochemical Accumulation and Yield in the Inflorescences of Hemp Plants

From the results obtained in the present study, it emerges that the inflorescences of unfertilized plants accumulated the lowest amount of phytochemicals compared to fertilized treatments. This gap increased even more when considering the phytochemical yield per plant due to the lower accumulation of dried biomass, which also characterized the inflorescences in F0. This result is not surprising as adequate nitrogen fertilization is required not only for sustaining plant growth but also for promoting the accumulation of secondary metabolites. In this regard, evidence suggests that dried biomass accumulation in the inflorescences, as well as CBD and THC concentration and yield, increased with the increasing nitrogen rate up to a peak of around 110 kg ha^−1^ [26,27]. In line with these findings, the results obtained in the present study revealed that the inflorescences in treatment NPK accumulated a total phytochemical content that was 44% higher than F0, and the yield was even higher (+191%) due to the accumulation of dried biomass in the inflorescences, which, in NPK, was more than twice that of F0.

As for combined mineral and organic fertilization, the results obtained indicate that 50% replacement of inorganic nitrogen fertilizer with the six composts and the non-composted solid digestate determined an overall phytochemical accumulation and yield higher than F0. In particular, high yields of phenolic acids and flavonoids characterized all organic treatments, with the highest yields achieved in C1 and C5, which exceeded those of NPK. After cannabinoids, phenolic compounds are the second most abundant class of secondary metabolites in hemp inflorescences. They are known for their health-promoting effects, which include antioxidant, anti-inflammatory, cardioprotective, neuroprotective, antiviral, and anticancer properties [36,37]. Except for C6, all organic treatments also showed high yields of carotenoids and tocopherols, which, in C1 and C5, reached values comparable to those observed in NPK. Carotenoids and tocopherols are the two most abundant lipid-soluble antioxidants in plants, and they are known to play a key role in the prevention of several human diseases [38,39]. Although the amounts detected for these compounds in hemp inflorescences are not so high as in other crops, they can still have an impact on the health properties of hemp inflorescences thanks to their synergistic action with phenolic compounds, which can strengthen their bioactivity, thereby reducing the required doses for producing beneficial effects [40]. In light of this, we can assert that the inflorescences obtained by replacing 50% inorganic nitrogen fertilizer with composts from C1 to C5 might be an effective source of phenolic compounds, carotenoids, and tocopherols, which are increasingly being used as ingredients in a variety of food, nutraceutical, and cosmetic formulations.

All organic treatments, except C6 and SD, also accumulated high levels of terpenes and cannabinoids, with the highest yields observed in C1, C2, and C3, where they achieved yields comparable or even higher than those observed in NPK. The high yields of terpenes are particularly interesting when hemp inflorescences are used to extract the essential oil, a hemp product that finds application in the agrochemical sector for the development of eco-friendly biopesticides [41], as a scent in cosmetics, and as an ingredient in body care products due to its emollient and hydrating properties [3]. Purification of specific molecules, i.e., β-caryophyllene, α-humulene, β-curcumene, and α-bisabolol, which are used as food and cosmetic additives or for their potential therapeutic applications [42,43,44], can also be achieved from these inflorescences. On the other hand, the high CBD yield is of significant interest for the pharmaceutical industry thanks to the wide range of health properties attributed to this molecule, which include, but are not limited to, antiepileptic, neuroprotective, anxiolytic, antipsychotic, anti-inflammatory, analgesic, and anticancer properties [45].

## 4. Materials and Methods

### 4.1. Preparation and Analysis of the Six Composts and the Digestate

The preparation of the six composts (C1–C6) used in the present study was carried out at the Research Centre for Cereal and Industrial Crops, Caserta, Italy. The raw materials and the proportions used for the preparation of each compost are reported in Table 4.

Cardoon waste was provided from a farm in Sardinia and was supplied by Novamont (Novamont S.p.A.—Via G. Fauser 8, 28100 Novara, Italy), spent mushroom substrates were from Bioagritest s.r.l. (Pignola, Potenza, Italy), and solid digestate from buffalo effluent was provided by the Power Rinasce S.p.A. anaerobic digestion plant (Santa Maria La Fossa, Caserta, Italy). The composting process lasted 132 days and consisted of a thermophilic phase of 20 days, with temperatures between 48 °C and 55 °C, a mesophilic (30–48 °C), and a slow maturation phase (25 °C) of 112 days.

The solid digestate from buffalo effluent used to prepare the six composts was also used alone in its non-composted form (SD).

The six composts and the non-composted solid digestate were sieved to 2 mm and ground to 0.5 mm by a mill (A11 basic, IKA, Staufen, Germany) and their main physicochemical properties were analyzed. pH and electrical conductivity (EC, dS m^−1^) were measured in a water-soluble extract (1:10 *w*/*v*) using a conductivity/pH meter Crison, (Hach Lange, Barcelona, Spain). Organic carbon (OC, g Kg^−1^) was determined by wet combustion with a mixture of potassium dichromate and sulfuric acid and measured through titration (Mettler Toledo DL28 Tritator, Mettler-Toledo S.p.A., Milano, Italy) [46]. The total nitrogen (N, g Kg^−1^) was determined by the Kjeldahl method using a semi-automated block digester (Heating Digestor DK, Velp Scientifica, Usmate, Monza Brianza, Italy) and continuous flow colorimetry (Autoanalyzer III, Bran Luebbe, Elmsford, NY, USA), measuring NH_4_^+^ through the Berthelot reaction [47]. The results obtained, reported in Table 5, were found to be in line with current Italian regulations [48].

### 4.2. Plant Material and Experimental Design

The dioecious hemp cv Eletta Campana, registered in the Italian/EU register of plant cultivars and characterized by a prevalence of CBD and a THC level below 0.3%, was used. The field trial was carried out at the experimental field of the Research Centre for Cereal and Industrial Crops of Caserta (lat. 41°04′26.1″ N; long. 14°19′05.5″ W), Caserta, Italy, in 2022.

Nine fertilization treatments were designed as follows: C1–C6, amendment with each of the six composts; SD, amendment with non-composted solid digestate from buffalo effluent; NPK, inorganic fertilization; F0, no fertilization. The six composts and the non-composted solid digestate were distributed (mixed into the topsoil, 25–30 cm) at a dose of 16 t ha^−1^. Before sowing, 100 kg ha^−1^ of K_2_O and 60 kg ha^−1^ of P_2_O_5_ were distributed in the NPK treatment. At side dressing (stem elongation phase, 9 June), nitrogen fertilizer (NH_4_NO_3_, 34% N) was applied at a rate of 50 Kg ha^−1^ in C1–C6 and SD treatments and at a rate of 100 Kg ha^−1^ in the NPK treatment, whereas no nitrogen fertilizer was applied for the F0 treatment.

**Figure 5:**
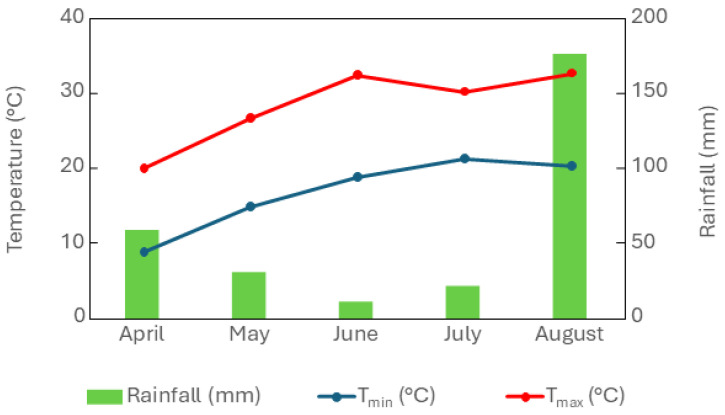
Weather parameters: rainfall (mm), minimum temperature (T_min_, °C), and maximum temperature (T_max_, °C) during the cropping season 2022.

The crop was hand-sown on 4 May using 40 kg ha^−1^ of seeds with an inter-row spacing of 20 cm. A randomized block experimental design with four replicates was used to distribute the nine treatments in the field, and each experimental unit had a surface area of 12 m^2^. Plants were drip-irrigated only if necessary. Manual weed control was used during the initial stages of crop development. Female plants were harvested at the phenological stage corresponding to full flowering, encoded as BBCH 67 [49]. During the experimental period, rainfall and minimum and maximum air temperature were registered, and the data are reported in Figure 5. The monthly means of maximum temperature were higher than 30 °C in June and August. Total rainfall was 300 mm, of which 177 mm fell in August.

### 4.3. Determination of Morphological Parameters and Biomass

For each replicate, six female plants were randomly collected and analyzed for the following parameters: plant height, basal and middle stem diameter, length of the main inflorescence, number of true leaves, fresh and dry weight of stem, inflorescence, leaves, and whole plant, with this latter parameter obtained as the sum of the weight of the individual organs. Plant height was measured as the distance from the plant base to the top of the main inflorescence, basal stem diameter was measured at 10 cm from the plant base, and middle stem diameter was measured halfway up the stem. For dry weight determination, fresh samples were placed in a ventilated oven at a temperature of 60 °C for 72 h until a constant weight was reached.

### 4.4. Spectrophotometric Measurements

#### 4.4.1. Preparation of the Extract

For each replicate, the inflorescences collected from the six female plants were pulverized into a fine powder using a planetary mill with jar balls (Pulverisette 7, Fritsch, Milan, Italy). Two grams of ground sample were extracted with 20 mL of a methanol–water mixture (80:20, *v*/*v*). The mixture was sonicated in an ultrasonic bath (Elmasonic P, Elma Schmidbauer GmbH, Singen, Germany) at 40 °C for 1 h in darkness. The suspension was then centrifuged at 10,000× *g* for 20 min at 4 °C, and the supernatant was analyzed daily.

#### 4.4.2. Determination of the Total Phenolic Content and Total Flavonoid Content

TPC and TFC were determined according to previously reported protocols, with minor modifications [50]. For the TPC assay, 100 μL of methanolic extract was mixed with 1100 μL of Folin–Ciocalteu reagent (10% *v*/*v*) and 800 μL of Na_2_CO_3_ solution (7.5% *w*/*v*). After 120 min incubation in the dark at room temperature, the absorbance at 765 nm was measured. For the TFC assay, 200 μL of methanolic extract was mixed with 1280 µL of ultrapure water and 60 µL of NaNO_2_ (5% *w*/*v*). After 5 min, the mixture was supplemented with 60 µL of AlCl_3_ (10% *w*/*v*) and, after another 5 min, 400 μL of 1 M NaOH. After 15 min incubation, the absorbance at 510 nm was measured. For each sample, the assay was carried out in triplicate using a Beckman DU64 UV/Vis Spectrophotometer (Indianapolis, IN 46268, USA). TPC was expressed as the mg of gallic acid equivalents for g^−1^ of dry weight (D.W.), whereas TFC was expressed as the mg of catechin equivalents g^−1^ D.W.

#### 4.4.3. Determination of the Total Antioxidant Activity

The total antioxidant activity of the methanolic extract was determined by the DPPH assay according to the protocol reported by Iannucci and coworkers [50]. A total of 6 mg of DPPH was added to a 100 mL methanol–water mixture (50:50, *v*/*v*) to prepare a DPPH radical solution with an absorbance value of 0.80 at 515 nm. The assay was carried out by adding 100 μL of the methanolic extract to 3900 μL of the DPPH radical solution. After 30 min incubation in the dark, the decolorization of the radical solution was evaluated by measuring the absorbance decrease at 515 nm. For each sample, the assay was carried out in triplicate using a Beckman DU64 UV/Vis Spectrophotometer (Indianapolis, IN 46268, USA). The results are expressed as the μmol of Trolox equivalent g^−1^ D.W.

### 4.5. HPLC Measurements

#### 4.5.1. Determinations of Free and Bound Phenolic Compounds

The extraction of phenolic compounds was performed according to Niño-Medina and coworkers [51], with minor modifications. For the extraction of the free phenolic fraction, 200 mg of the ground sample was resuspended in 3 mL of a methanol–water mixture (80:20, *v*/*v*). The suspension was purged for 30 s with argon, stirred for 2 h, and then centrifuged at 6000× *g* for 5 min at 25 °C. The supernatant was recovered and stored at −20 °C until use, whereas the pellet was supplemented with 5 mL of 2 M NaOH to perform the extraction of the bound phenolic fraction. The suspension was stirred for 2 h; then, its pH was adjusted to 2.5 via the addition of concentrated HCl. The extraction of bound phenolic compounds was performed by adding 5 mL diethyl ether to the suspension and centrifuging in at 6000× *g* for 5 min at 25 °C. The extraction was repeated twice, and the two supernatants recovered were pooled and evaporated to dryness before being reconstituted with a methanol–water mixture (80:20, *v*/*v*) and stored at −20 °C until use.

The phenolic compounds in the methanolic extracts were identified and quantified according to Kim and coworkers [52] using an HPLC system equipped with a diode-array detector (Agilent Technologies, Waldbronn, Germany). The separation was achieved using a Zorbax SB-C18 column 250 mm × 4.6 mm × 5 μm (Agilent, Santa Clara, CA, USA). The mobile phase consisted of 1% acetic acid in water (*v*/*v*) (solution A) and acetonitrile (solution B). The elution gradient was set as follows: 95% A, 0 min; 85% A, 30 min; 50% A, 40 min; 0 A%, 44 min; 95% A, 46 min; and an isocratic elution of 95% A, 46–50 min. The flow rate was 1 mL min^−1^. The column temperature was set at 35 °C, and the injection volume was 10 µL. Phenolic compounds were detected at 280 and 320 nm, and their identification and quantification were carried out by comparison with authentic standards (by retention time, spectrum similarity, and calibration curves). For each sample, the assay was carried out in triplicate, and the results are expressed as μg g^−1^ D.W.

#### 4.5.2. Determination of Carotenoids

A total of 200 mg of ground sample was extracted with 4 mL of water-saturated butan-1-ol. The suspension was incubated at room temperature for 1 h under continuous shaking and then centrifuged at 4000× *g* for 10 min at 8 °C. The extraction was repeated twice, and the two supernatants recovered were pooled and filtered through a 0.22 mm PTFE membrane (Millipore, Carrigtwohill Co., Cork, Ireland). The carotenoids were identified and quantified according to the protocol reported by Beleggia and coworkers [53], using the same HPLC system used for the determination of the phenolic compounds. The separation was performed using a C18 column (Synergi 4 μm Hydro RP 250 × 4.6 mm, Phenomenex, Torrance, CA, USA) and a C18 precolumn (4.0 × 3.0 mm, Phenomenex, Torrance, CA, USA). The mobile phase consisted of water (solution A) and acetone (solution B). The elution gradient was set as follows: 35% A, from 0 to 5.1 min; from 35% to 10% A, from 5.1 to 9.1 min; 10% A, from 9.1 to 11.9 min; from 10% to 0% A, from 11.9 to 13.7 min; 0% A, from 13.7 to 16.8 min; from 0% to 35% A, from 16.8 to 17.7 min; and 35% A as a post-run, from 17.7 to 23 min. The flow rate was 0.8 mL min^−1^. The column temperature was set at 30 °C, and the injection volume was 20 μL. Carotenoids were detected at 450 nm by a photodiode array detector (Agilent Technologies, Waldbronn, Germany), and their identification and quantification were carried out by comparison with authentic standards (by retention time, spectrum similarity, and calibration curves). For each sample, the assay was carried out in triplicate, and the results are expressed as μg g^−1^ D.W.

#### 4.5.3. Determination of Tocopherols

One hundred milligrams of ground sample were extracted with 3 mL acetonitrile. The suspension was incubated at room temperature for 30 min under magnetic stirring and then centrifuged at 3000× *g* for 15 min at 10 °C. The supernatant was dried by a centrifugal evaporator (Jouan RC 1022, Thermo Electron Corp., American Laboratory Trading, Inc., East Lyme, CT, USA), resuspended in 1 mL methanol, and filtered through a 0.22-mm PTFE membrane (Millipore, Carrigtwohill Co., Cork, Ireland). Tocopherols were separated and quantified according to the protocol reported by Beleggia and coworkers [53], using the same HPLC system used for the determination of the phenolic compounds. Acetonitrile/methanol/2-propanol (40:55:5, *v*/*v*/*v*) was used as the mobile phase under isocratic conditions. The flow rate was 0.8 mL min^−1^, and the total time of analysis was 30 min. The column temperature was set at 30 °C, and the injection volume was 20 μL. Tocopherols were detected by a fluorescence detector (excitation at 292 nm and emission at 335 nm), and their identification and quantification were carried out by comparison with authentic standards (i.e., by retention time, spectrum similarity, and calibration curves). The β-tocopherol and γ-tocopherol eluted together; therefore, they are presented as the sum of both isomers. For each sample, the assay was carried out in triplicate, and the results are expressed as μg g^−1^ D.W.

### 4.6. Determination of Terpenes and Cannabinoids by GC-MS

Non-polar secondary metabolites were extracted essentially as reported by Beleggia and coworkers [54], using an accelerated solvent extractor (Dionex ASE350, Thermo Fisher Scientific Inc., Waltham, MA, USA). Two hundred milligrams of ground sample were placed in a stainless steel cell, supplemented with 10 μL of the internal standard (pentadecane, 11.5 mg/mL), and extracted with 5.5 mL n-hexane. A single extraction cycle was performed using the following extraction conditions: 50 °C, 10 MPa, 5 min heating, and 15 min static extraction. The extract obtained (1 μL) was injected into the GC-MS system (Agilent 6890A coupled to a Triple Quadrupole mass Spectrometer 7000B, Agilent Technologies, Santa Clara, CA, USA), equipped with an HP-5ms capillary column (30 m × 0.25 mm i.d. × 0.25 μm film thickness), and the separation was carried out following the procedure reported by Beleggia and coworkers [54], with minor modifications. The oven temperature program was set at 60 °C for 1 min, followed by an increase of 8 °C min^−1^ up to 220 °C, maintained for 1 min, and an 8 °C min^−1^ increase up to 280 °C, maintained for 25 min. The injection and the transfer line temperatures were set at 280 °C, and the source temperature was set at 240 °C. Helium was used as a carrier gas at a constant flow of 1 mL min^−1^. The acquisition was conducted in full scan, with a 50–700 *m*/*z* scan range. The identification of metabolites was based on the comparison of their mass spectra with those of authentic standards, or by the matching them against the commercial mass spectra library NIST 11, and it was confirmed by comparison of their linear retention indices relative to the series of n-hydrocarbons. Semi-quantification was achieved via peak normalization with those of the internal standard added and the sample weight. For each sample, the assay was carried out in triplicate, and the results are expressed as μg g^−1^ D.W.

### 4.7. Statistical Analysis

JMP software version 8.0 (SAS Institute Inc., Cary, NC, USA) was used to estimate significant differences among the means through Tukey’s multiple test (*p* ≤ 0.05). The MetaboAnalyst 6.0 online tool (https://www.metaboanalyst.ca) was used to perform the heatmap associated with HCA. To perform the analysis, all data were auto-scaled, i.e., mean-centered and normalized to the standard deviation. A dendrogram was constructed on Euclidean distance using Ward’s method.

## 5. Conclusions

Overall, the picture that emerges from the present study reveals that while the replacement of 50% mineral nitrogen with composts from C1 to C5 was successful in promoting the growth and development of hemp plants, it also induced phytochemicals to accumulate in their inflorescences. As a result, these treatments are suitable for hemp cultivation intended for the production of biomass or fiber, as well as for the extraction of phytochemicals from the inflorescence. Furthermore, the ability of these composts to stimulate the accumulation of the various classes of phytochemicals differently offers the opportunity to generate customized phytochemical profiles that fulfill the needs of specific industrial applications. In this regard, future studies are needed to elucidate the mechanisms underlying the different changes in the hemp inflorescence secondary metabolism induced by the application of the different composts. As with other crops, using a combination of organic and inorganic fertilization in hemp cultivation will help to reduce the risks of environmental pollution derived from nitrogen volatilization and lixiviation while also improving the soil’s physicochemical and microbiological characteristics, as well as the recycling of organic waste.

## Figures and Tables

**Table 1:** Effect of the different treatments on hemp plant height, stem diameter, inflorescence length, and number of leaves per plant.

Treatment ^a^	Plant Height (cm)	Basal Stem Diameter (mm)	Middle Stem Diameter (mm)	Inflorescence Length (cm)	Number of Leavesper Plant (n)
C1	223.6 abc	12.2 ab	10.0 ab	55.9 a	17.9
C2	224.3 abc	11.6 abc	9.6 ab	45.1 ab	18.3
C3	218.3 abc	11.2 abc	9.2 abc	50.3 ab	16.7
C4	227.1 ab	11.8 abc	9.5 ab	50.7 ab	17.3
C5	254.7 a	13.0 a	10.8 a	57.8 a	15.4
C6	184.5 c	10.4 bc	8.8 bc	44.4 ab	15.6
SD	199.2 bc	10.9 abc	8.9 abc	45.1 ab	17.1
NPK	237.8 a	12.5 ab	10.1 ab	53.8 a	16.3
F0	201.4 bc	9.6 c	7.6 c	38.5 b	16.3

^a^ C1–C6, composts 1–6; SD, non-composted solid digestate; NPK, inorganic fertilization; F0, no fertilization. For each trait different lower-case letters represent significant differences among treatments (Tukey’s test *p* ≤ 0.05).

**Table 2:** Effect of the different treatments on the abundance of the individual metabolites detected in the hemp inflorescences.

Class	Metabolite ^b^	Treatment ^a^								
		C1	C2	C3	C4	C5	C6	SD	NPK	F0
Flavonoids	Free vitexin	1435.1 cd	1279.8 d	821.9 e	1627.0 bc	2112.3 a	1747.2 b	1383.7 cd	1825.9 b	449.3 f
	Free isovitexin	1202.0 ab	1283.5 ab	1273.5 ab	1181.7 ab	1321.9 a	1253.7 ab	1346.8 a	1078.9 b	405.4 c
	Free luteolin	88.1 b	116.9 a	84.0 b	128.2 a	84.6 b	47.6 c	66.4 bc	81.0 b	46.4 c
	Free orientin	985.1 c	1009.1 c	1269.8 b	657.0 d	1040.3 c	1051.6 c	1576.8 a	1017.0 c	569.2 d
	Free apigenin	58.1	68.3	82.5	57.9	35.5	45.5	45.7	49.8	41.6
	Bound isovitexin	48.2 a	29.5 cd	18.0 d	26.8 cd	44.5 ab	32.5 bc	47.0 a	35.1 abc	32.5 bc
	Bound luteolin	14.2 ab	17.9 a	10.4 bc	12.8 b	10.2 bc	6.3 c	11.4 b	9.6 bc	6.5 c
	Bound apigenin	8.5 bcd	11.3 ab	12.1 a	9.5 abc	6.0 d	6.0 d	6.8 cd	9.1 abcd	6.8 cd
Phenolic acids	Free *p*-hydroxybenzoic acid	80.5	91.3	85.5	82.8	93.8	84.4	64.6	71.5	91.5
	Free caffeic acid	37.9	28.0	35.2	29.6	50.7	32.9	43.0	37.0	49.0
	Free *p*-cumaric acid	152.6 ab	95.0 e	121.8 cd	102.2 de	172.5 a	135.5 bc	132.7 bc	119.7 cd	117.5 cde
	Free ferulic acid	39.8 ab	27.1 b	36.6 ab	58.3 ab	105.2 a	65.5 ab	98.4 ab	53.3 ab	18.6 b
	Bound *p*-hydroxybenzoic acid	9.2	11.3	10.1	8.7	9.3	9.2	7.4	6.9	9.2
	Bound caffeic acid	3.0 d	8.7 c	8.0 c	6.3 cd	13.7 b	15.9 ab	19.0 a	13.1 b	2.3 d
	Bound *p*-cumaric acid	175.9 a	114.4 c	76.9 d	98.2 cd	149.0 ab	122.3 bc	160.5 a	114.3 c	74.7 d
	Bound ferulic acid	28.2 a	22.6 a	11.3 b	21.1 a	24.2 a	22.4 a	26.0 a	20.4 ab	19.2 ab
Carotenoids	Lutein	212.2 ab	148.7 de	158.7 cde	177.2 bcd	214.4 a	124.4 e	185.5 abc	210.6 ab	152.6 cde
	Zeaxhantin	43.9	33.8	36.1	37.1	38.4	34.5	35.0	38.7	32.6
	β-Carotene	141.5 a	96.6 cde	104.8 bcd	121.0 abc	143.9 a	81.9 de	127.4 abc	129.9 ab	64.9 e
Tocopherols	δ-Tocopherol	1.3	1.3	1.6	1.1	1.5	1.6	1.7	1.5	1.2
	β + γ-Tocopherol	19.0	18.7	19.5	22.7	19.2	17.7	20.9	24.2	18.3
	α-Tocopherol	153.6 b	138.8 b	144.3 b	142.7 b	227.8 a	94.3 bc	221.8 a	238.2 a	64.8 c
Monoterpenes	α-Pinene	6.1 ab	13.3 a	5.8 ab	3.4 b	5.6 ab	5.3 ab	1.7 b	4.5 b	2.3 b
	β-Pinene	2.1	2.3	0.6	1.8	1.9	1.2	0.7	1.6	0.6
	β-Myrcene	0.7 a	0.9 a	0.7 a	0.7 a	1.2 a	0.7 a	0.0 b	0.0 b	0.0 b
	Limonene	1.0 abc	1.7 a	1.4 ab	1.5 ab	0.9 bc	0.9 bc	0.6 c	0.9 bc	0.6 c
	*p*-Cymene	5.8	6.6	5.6	6.2	4.7	4.7	4.3	5.9	5.3
Oxygenated monoterpenes	Eucalyptol	2.0	1.6	2.6	2.7	2.8	1.8	2.4	2.5	1.9
	Terpinen-4-ol	1.0 bc	2.1 a	1.3 ab	2.1 a	0.0 c	1.7 ab	1.0 b	1.0 b	1.3 ab
	α-Terpineol	0.8	1.1	1.0	1.1	0.7	0.8	0.7	1.2	0.8
Sesquiterpenes	α-Ylangene	0.6 c	2.4 ab	2.7 a	1.9 abc	1.1 bc	1.9 abc	1.0 bc	1.0 bc	1.5 abc
	β-Caryophyllene	156.5	147.0	158.7	127.0	107.9	126.9	52.6	116.0	160.2
	Patchoulene	1.4 b	3.2 ab	5.4 a	1.1 b	0.5 b	1.9 b	1.2 b	2.7 ab	1.5 b
	α-Humulene	137.2	128.2	154.7	110.6	88.6	118.9	75.5	103.1	145.5
	Aromadendrene	4.4 bc	6.1 b	19.9 a	3.0 cd	2.0 d	2.5 cd	2.1 d	2.6 cd	2.8 cd
	β-Selinene	0.5	1.8	2.2	1.4	1.9	1.7	1.3	1.5	1.9
	β-Guaiene	1.4 b	6.0 ab	8.4 a	3.3 b	2.6 b	6.6 ab	2.1 b	4.4 ab	3.9 b
	α-Selinene	7.7	13.1	13.7	9.0	4.6	7.7	4.9	8.9	10.3
	β-Curcumene	128.9	109.6	125.6	118.2	126.9	118.5	114.5	111.9	122.5
	Cubenene	2.6 b	8.5 ab	15.4 a	3.0 b	3.0 b	8.1 ab	3.1 b	3.1 b	4.4 b
	δ-Cadinene	5.0 b	13.0 ab	18.7 a	7.8 b	6.5 b	10.4 ab	4.5 b	6.8 b	10.2 ab
	α-Muurolene	7.0 bc	33.5 a	27.5 a	0.0 c	0.0 c	11.5 b	6.8 bc	8.8 bc	0.0 c
	Selina-3,7(11)-diene	23.7 abc	23.1 abcd	28.5 abc	30.0 ab	40.3 a	11.2 bcd	8.8 d	9.8 cd	26.9 abcd
Oxygenated sesquiterpenes	Selina-6-en-4-ol	2.6 b	10.9 a	5.3 ab	3.9 b	3.9 b	2.7 b	2.4 b	2.8 b	2.8 b
	*trans*-Longipinocarveol	8.6 abc	16.3 a	14.2 ab	10.1 abc	7.0 bc	7.3 bc	5.7 c	7.2 bc	10.0 abc
	Longifolenaldehyde	5.8 ab	7.8 a	7.5 a	4.5 bc	3.9 bc	4.3 bc	2.7 c	6.4 a	3.9 bc
	Alloaromadendrene oxide	5.1 b	10.7 a	8.7 a	4.4 b	3.4 b	3.7 b	3.1 b	4.5 b	3.7 b
	α-Bisabolol	137.9 a	118.3 ab	22.4 d	21.8 d	43.2 d	87.9 bc	53.5 cd	115.6 ab	46.8 d
	Eudesm-7(11)-en-4-ol	4.2 bc	12.8 a	7.5 b	6.6 b	4.3 bc	4.6 bc	2.2 c	4.4 bc	3.6 bc
	Clovanediol	5.2 a	6.2 a	5.9 a	4.3 a	0.5 b	0.5 b	0.5 b	0.7 b	0.5 b
Cannabinoids	Cannabidivarin	510.8 d	1432.8 bcd	3521.7 abc	1664.2 bc	725.4 d	1730.4 bc	950.9 cd	1904.9 b	1136.6 bcd
	Cannabidiol (CBD)	15,793.4 ab	16,370.8 a	18,099.5 a	15,953.4 ab	13,197.5 c	13,958.8 bc	12,770.6 c	16,669.4 a	13,004.1 c
	∆^9^-Tetrahydrocannabinol (THC)	392.1 ab	497.3 a	555.9 a	401.2 ab	327.7 bc	330.0 bc	192.2 c	184.8 c	192.4 c
	Cannabigerol (CBG)	312.0 a	260.6 ab	238.0 ab	159.9 bc	337.7 a	369.5 a	95.4 c	290.6 ab	51.9 c
	Cannabinol (CBN)	357.9	456.0	515.3	418.7	302.1	356.1	253.6	383.9	258.9

^a^ The metabolite content was expressed as μg g^−1^ of dry weight. ^b^ C1–C6, composts 1–6; SD, non-composted solid digestate; NPK, inorganic fertilization; F0, no fertilization. For each metabolite, different lower-case letters represent significant differences among treatments (Tukey’s test *p* ≤ 0.05).

**Table 3:** Effect of the different treatments on the yield per plant of the different classes of metabolites detected in the hemp inflorescences.

Class of Metabolites ^a^	Treatment ^b^								
	C1	C2	C3	C4	C5	C6	SD	NPK	F0
Total free and bound phenolic acids	5.96 ab	4.20 c	3.39 cd	4.05 c	6.73 a	3.77 c	4.32 bc	4.99 bc	2.13 d
Total free and bound flavonoids	43.44 abc	40.21 abc	33.21 c	35.83 bc	50.82 a	32.74 c	37.23 bc	47.64 ab	9.49 d
Total carotenoids	4.57 a	3.01 bc	2.73 bcd	3.28 ab	4.30 a	1.84 cd	2.88 bc	4.43 a	1.50 d
Total tocopherols	1.93 b	1.72 b	1.49 bc	1.64 b	2.78 a	0.88 cd	1.93 bc	3.04 a	0.45 d
Total monoterpenes and oxygenated monoterpenes	0.25 ab	0.34 a	0.17 bcde	0.19 bcd	0.19 bcd	0.12 cde	0.09 de	0.22 bc	0.08 e
Total sesquiterpenes and oxygenated sesquiterpenes	7.69 a	7.34 ab	6.07 abc	4.48 cd	4.86 bcd	4.17 cd	3.01 d	6.62 abc	3.33 d
Total cannabinoids	196.16 abc	200.26 ab	213.04 ab	183.05 abc	163.35 bcd	128.63 cde	116.44 de	229.61 a	84.90 e

^a^ For each class of metabolites, the yield was calculated by multiplying the concentration by the dried biomass of the inflorescence and was expressed as mg plant^−1^. ^b^ C1–C6, composts 1–6; SD, non-composted solid digestate; NPK, inorganic fertilization; F0, no fertilization. For each class of metabolites, different lower-case letters represent significant differences among treatments (Tukey’s test *p* ≤ 0.05).

**Table 4:** Composition of piles for composting.

Raw Material	Compost Composition (% *w*/*w*)					
	C1	C2	C3	C4	C5	C6
Solid digestate from buffalo effluent	50	50	50	67	75	84
Cardoon-based spent mushroom substrate	50	—	—	—	—	—
Straw-based spent mushroom substrate	—	50	—	—	—	—
Cardoon waste	—	—	50	33	25	16

**Table 5:** Main physicochemical properties of the six composts (C1–C6) and the non-composted solid digestate (SD).

Amendment	Property ^a^			
	pH	EC (dS m^−1^)	OC (g Kg^−1^)	N (g Kg^−1^)
C1	8.1 ± 0.00	1.11 ± 0.02	187.0 ± 0.03	18.4 ± 0.50
C2	8.0 ± 0.00	1.20 ± 0.07	190.8 ± 0.25	16.8 ± 0.50
C3	7.9 ± 0.07	0.84 ± 0.03	211.2 ± 0.13	6.4 ± 0.42
C4	7.9 ± 0.07	0.79 ± 0.01	214.6 ± 2.13	18.1 ± 0.96
C5	7.6 ± 0.07	0.96 ± 0.09	296.8 ± 4.98	21.6 ± 4.96
C6	7.8 ± 0.07	0.97 ± 0.08	282.2 ± 6.28	21.9 ± 1.70
SD	8.9 ± 0.07	1.11 ± 0.02	409.1 ± 1.34	17.6 ± 1.21

^a^ Values are expressed as mean ± standard deviation. EC, electrical conductivity; OC, total organic carbon; N, total nitrogen.

## Data Availability

The dataset is available on request from the authors.
